# Prognostic and predictive value of metastatic lymph node ratio in stage III gastric cancer after D2 nodal dissection

**DOI:** 10.18632/oncotarget.19998

**Published:** 2017-08-07

**Authors:** Yinbo Chen, Cong Li, Yian Du, Qi Xu, Jieer Ying, Cong Luo

**Affiliations:** ^1^ Department of Colorectal Surgery, Zhejiang Cancer Hospital, Hangzhou, Zhejiang 310022, P.R. China; ^2^ Department of Lymphoma Oncology, Zhejiang Cancer Hospital, Hangzhou, Zhejiang 310022, P.R. China; ^3^ Department of Abdominal Surgery, Zhejiang Cancer Hospital, Hangzhou, Zhejiang 310022, P.R. China; ^4^ Department of Abdominal Oncology, Zhejiang Cancer Hospital, Hangzhou, Zhejiang 310022, P.R. China

**Keywords:** adjuvant chemotherapy, gastric cancer, lymph node ratio

## Abstract

**Introduction:**

This study is to evaluate the prognostic and predictive value of metastatic lymph node ratio (MLR) in stage III gastric cancer following radical D2 dissection.

**Methods:**

87 patients who underwent radical resection with D2 lymphadenectomy were retrospectively evaluated. The median age was 60 with a 2:1 ratio of male/female. Of these 87 patients, 83 underwent total gastrectomy, the remaining 4 underwent subtotal gastrectomy and 57 patients received adjuvant chemotherapy with fluoropyrimidines. Indexes of lymph node involvement and other clinicopathological data were analyzed. Survival was determined by the Kaplan-Meier method and log-rank test. Multivariate analysis was performed using the Cox proportional hazards model.

**Results:**

Median total retrieved lymph node number was 35 (range: 10-104) with median metastatic lymph node amount of eight (range: 0-71). Median survival time was 31.7 months with a 3-year survival rate of 36.4%. Patients were divided into four groups according to MLR: MLR0, 0; MLR1, <0.1; MLR2, 0.1-0.25; MLR3, >0.25. After median follow-up of 31 months, median OS rates of MLR0 to MLR3 were 37.1m, 35.9m, 31.5m and 20.8m, respectively (p=0.013). Median OS rates were significantly different among subgroups: 39.3m and 36.5m were obtained for low subgroups (MLR<0.24) with or without adjuvant chemotherapy, respectively; 22.9m and 12.2m were found in high subgroups (MLR>0.24) with and without chemotherapy, respectively (p=0.002). Finally, MLR constituted an independent prognostic factor in multivariable analysis.

**Conclusions:**

After R0 resection with D2 lymphadenectomy for stage III gastric cancer, MLR constitutes an effective prognostic indicator. Patients with high MLR may benefit the most from adjuvant chemotherapy.

## INTRODUCTION

Gastric cancer is a common malignancy, being the fourth most common diagnosed cancer worldwide and accounting for 738,000 deaths each year placing it second in cancer deaths following lung cancer [1]. Even though TNM staging classification has been proved to be a simple, reliable and objective method for predicting the prognosis of gastric cancer, it still has some problems such as heterogeneous survival. Lymph node metastasis is one of the most important prognostic factors for gastric cancer patients who undergo radical resection [2, 3]. Yet to date, there is still lacking the best classification of N stage to predict the prognosis of gastric cancer [4]. A new version of the Japanese Classification for gastric cancer and treatment guidelines ( Japanese Gastric Cancer Association (JGCA)) redefined the N stage based on the level of lymph node involvement which was in accordance with the International Federation of cancer and the American Cancer Society (UICC/AJCC). Even in patients with D2 radical resection around the world, there still exists big difference in the number of lymph nodes examined [5]. With the increasing tested number of lymph nodes, the number of metastatic lymph nodes also increases. Lymph node ratio(MLR) is defined as number of metastatic lymph nodes divided by total resected lymph nodes, which is a supplement to N staging, especially in patients with lymph nodes examined less than 15. Two large-scale studies found AJCC staging misclassified 57% of patients while the MLR misclassified only 12%, suggesting MLR was surperior over the present N staging system about the potential for stratification of gastric cancer patients [6, 7]. The application of MLR was showed to be a good prognostic factor in gastric cancer in the retrospective analyses [8-13]. However, few previous studies analyzed the prognostic value of MLR in stage III gastric cancer, neither did they evaluate the association between MLR and adjuvant chemotherapy. Considering the high incidence of stage III gastric cancer in Chinese patients and its heterogeneity in survival, thus, the aim of this study was to evaluate the prognostic significance of MLR, and to investigate whether MLR was an appropriate predicting factor for adjuvant chemotherapy in stage III gastric cancer after D2 dissection.

## RESULTS

### Clinicopathological characteristics of patients

A total of 87 patients were included in the present study. The median age of patients was 60 (range: 21 to 83) with 58 males (66.7%) and 29 females (33.3%). The surgical modalities were as following: 82 total gastrectomy (94.3%), five subtotal gastrectomy (5.7%). Of those 87 patients, 57(65.5%) received chemotherapy, including neoadjuvant chemotherapy in 19 patients. All chemotherapy regimens contained Flouropyrimidines (5-FU) or capecitabine. Only five patients received flouropyrimidines single-agent, while 52 patients received combined-chemotherapy (Table 1).

**Table 1:** Clinical and pathological characteristics of 87 patients and univariate analysis

Variable		No. of patient (%)	OS(months)	P value
Age (years)	≤60	44 (50.6)	39.2	0.115
	>60	43 (49.4)	28.6	
Gender	Male	58 (66.7)	30.6	0.565
	Female	29 (33.3)	31.8	
Differentiated degree	G1	3 (3.4)	32.2	0.214
	G2	32 (36.8)	36.4	
	G3	52 (59.8)	29.5	
Tumor localization	Cardias	15 (17.2)	30.6	0.079
	Body/fundus	10 (11.5)	22.5	
	Antrum/pylorus	35 (40.2)	36.4	
	Diffuse	27 (31.0)	17.2	
Borrmann type	I	3 (3.4)	33.5	0.799
	II	0 (0)	-	
	III	62 (71.3)	31	
	IV	22 (25.3)	31.8	
Depth of invasion	T1	0		0.161
	T2	2 (2.3)	22.8	
	T3	17 (19.5)	36.4	
	T4	68 (78.2)	29.5	
HER-2 IHC	0	34(39.1)	31	0.134
	+	34(39.1)	27.9	
	++	9(10.3)	22.5	
	+++	2(2.3)	36.5	
	unknown	8(9.2)	49	
Lymph node stage	N0	5 (5.7)	37.1	0.012
	N1	17 (19.5)	36.4	
	N2	16 (18.4)	30.6	
	N3a	26 (29.9)	31.8	
	N3b	23 (26.4)	19.7	
Disease stage	IIIa	8 (9.2)	35.9	0.029
	IIIb	20 (23.0)	39.2	
	IIIc	59 (67.8)	20.8	
MLR	MLR0	5(5.7)	37.1	0.013
	MLR1	24(27.6)	35.9	
	MLR2	15(17.2)	31.5	
	MLR3	43(49.4)	20.8	

### Nodal status distribution and node ratio categories

All the patients received extensive lymphadenectomy except two of them were resected lymph nodes less than 15 (MLR was 0.9 and 0.85). The median number of removed lymph nodes was 35 (range: 10 to 104) and the median number of metastatic lymph nodes was 8 (range: 0 to 71). In brief, patients were classified into metastatic lymph nodes ratio MLR0 to MLR3, based on the following intervals: MLR0: 0%; MLR1:≤10%; MLR2: 11%-25%; MLR3: >25%. The median MLR was 24%.

MLR had a significant correlation with tumor differentiated degree and disease stage. MLR3 was found significantly more common in patients with G3 (p=0.033) or with IIIC stage (p<0.001).Meanwhile, each N category was stratified into different MLR subgroups. The results indicated a good consistency of the two classification methods: as the increase of N stage, MLR increased (Table 2).

**Table 2:** The correlation between MLR and clinicopathological factors

Factor		No. of patient	MLR				Chi-square P-value
			0	1	2	3	
Differentiated	G1	3	1(33.3%)	1(33.3%)	0(0%)	1(33.3)	0.033
degree	G2	32	4(12.5%)	11(34.4%)	6(18.8%)	11(34.4%)	
	G3	52	0(0%)	12(23.1%)	9(17.3%)	31(59.6%)	
Tumor stage	T1-3	19	0(0%)	8(42.1%)	4(21.2%)	7(36.8%)	0.237
	T4	68	5(7.4%)	16(23.5%)	11(16.2%)	36(52.9%)	
Tumor location	Cardia	15	1(6.7%)	4(26.7%)	4(26.7%)	6(40%)	0.363
	Body	10	2(20%)	0(0%)	2(20%)	6(60%)	
	Antropyluric	35	1(2.9%)	10(28.6%)	6(17.1%)	18(51.4%)	
	Diffuse	27	1(3.7%)	10(37%)	3(11.1%)	13(48.1%)	
Borrmann type	I	3	0(0%)	2(66.7%)	0(0%)	1(33.3%)	0.599
	II	0					
	III	62	3(48%)	16(25.8%)	13(21%)	30(48.4%)	
	IV	22	2(9.1%)	6(27.3%)	2(9.1%)	12(54.5%)	
Disease stage	IIIA	8	0(0%)	6(75%)	0(0%)	2(25%)	<0.001
	IIIB	20	5(25%)	14(70%)	1(5%)	0(0%)	
	IIIC	59	0(0%)	4(6.8%)	14(23.7%)	41(69.5%)	
N stage	N0	5	5(100%)	0(0%)	0(0%)	0(0%)	<0.001
	N1	17	0(0%)	17(100%)	0(0%)	0(0%)	
	N2	16	0(0%)	7(43.8%)	8(50%)	1(6.3%)	
	N3a	26	0(0%)	0(0%)	7(26.9%)	19(73.1%)	
	N3b	23	0(0%)	0(0%)	0(0%)	23(100%)	

### Survival analysis

**Figure 1:**
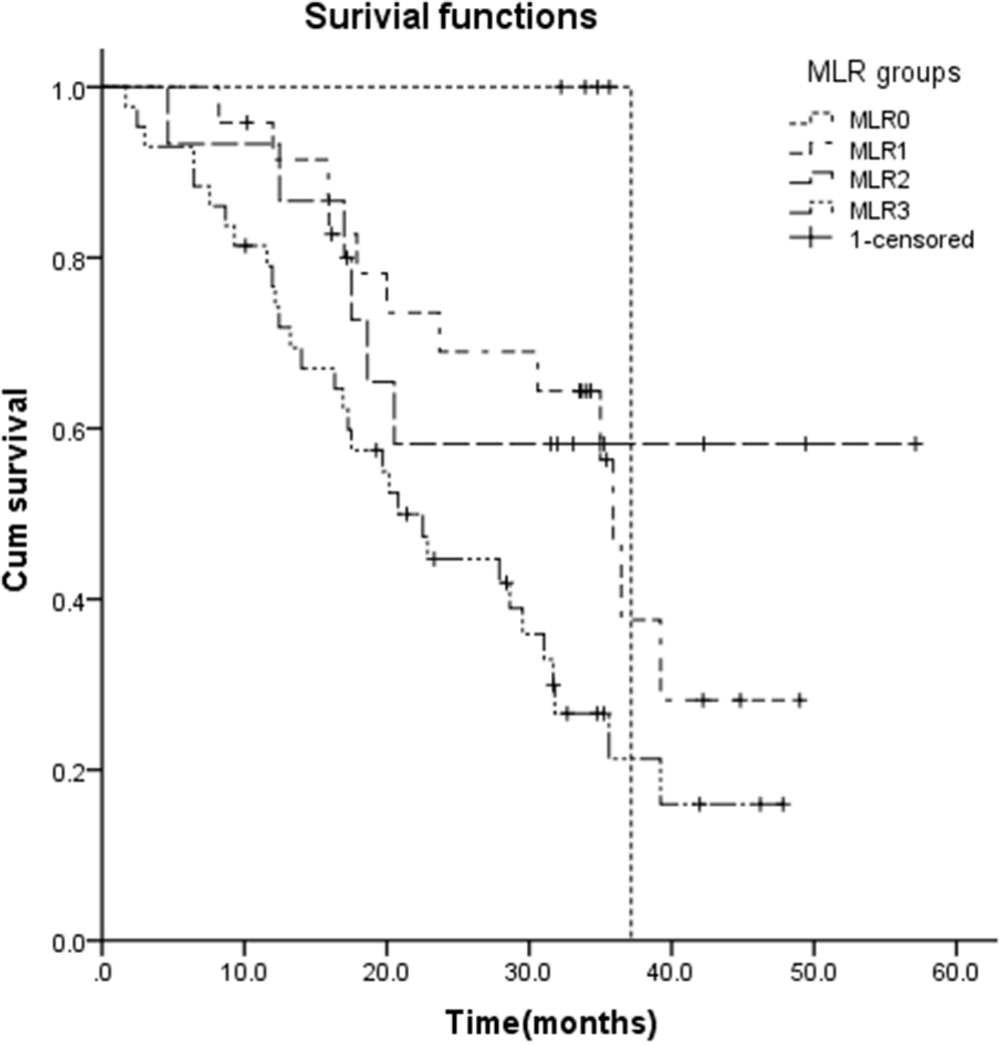
The overall survival in different MLR groups: OS of the MLR0 to MLR3 categories was 37.1m, 35.9m, 31.5m and 20.8m, respectively (p=0.013)

After a median follow-up time of 31 months (range:2 to 57months), 50 patients (57.5%) succumb to the disease. The median DFS was 24.8 months and median (OS) was 31.7 months. The overall 3-year survival rate was 36.4%. OS of the MLR0 to MLR3 categories was 37.1m, 35.9m, 31.5m and 20.8m, respectively (p=0.013) (Figure 1).

As shown in Table 2, factors including age, gender, tumor stage, tumor location, Borrmann type, depth of invasion, lymph node stage, disease stage and MLR were analyzed in univariate analysis. Disease stage, lymph node stage and MLR have significant impact on survival. In multivarariate analysis, MLR has remained an independent prognostic factor of survival (Hazard ratio: 1.47, 95% CI: 1.014-2.120, P=0.042).

**Figure 2:**
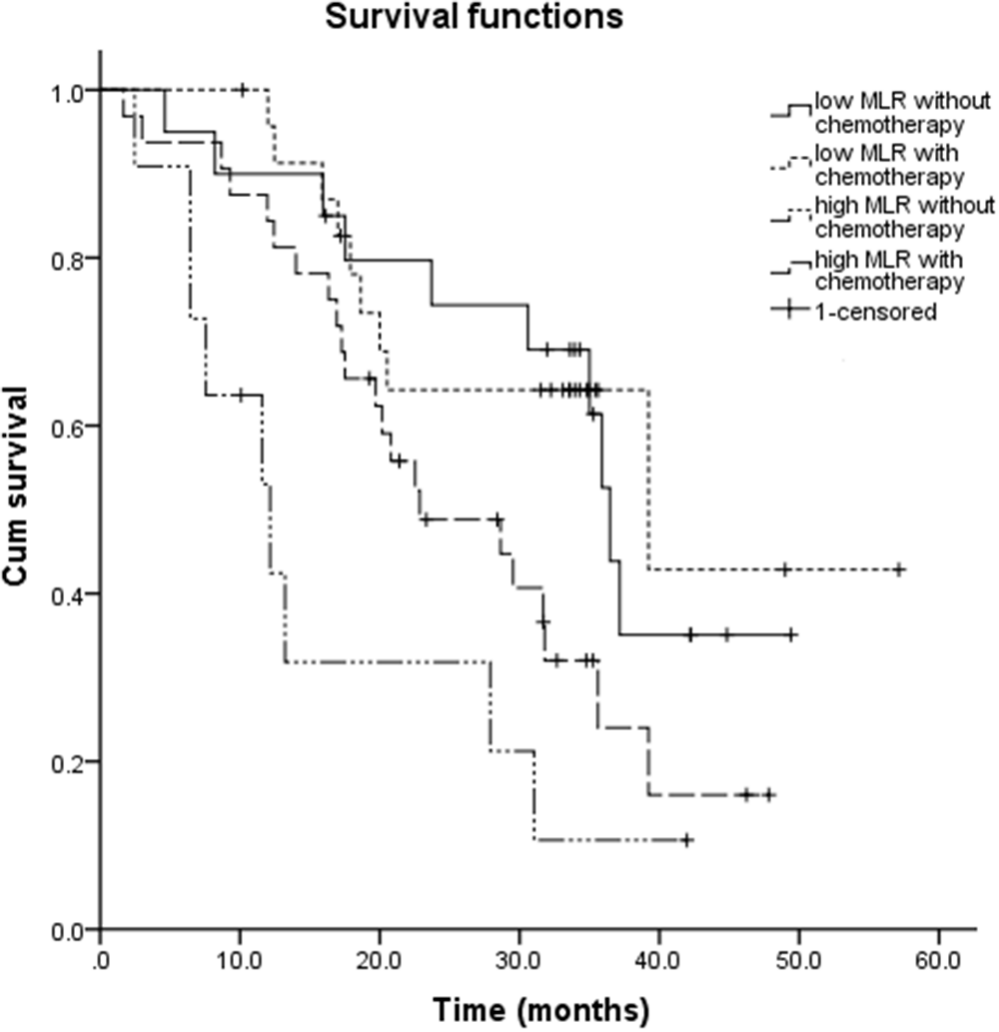
The correlation of MLR with adjuvant chemotherapy: OS of patients in low MLR group without chemotherapy was 36.5 months, OS of patients in low MLR group with chemotherapy was 39.3 months, OS of patients in high MLR group without chemotherapy was 12.2 months, and OS of patients in high MLR group with chemotherapy was 22.9 months, respectively (p=0.002)

We further analyzed the correlation of MLR with adjuvant chemotherapy. When stratifying patients into low MLR (MLR≤24%) or high MLR (>24%) subgroups, significant differences of survival were found between patients with adjuvant chemotherapy or not: OS of patients in low MLR group without chemotherapy was 36.5 months, OS of patients in low MLR group with chemotherapy was 39.3 months, OS of patients in high MLR group without chemotherapy was 12.2 months, and OS of patients in high MLR group with chemotherapy was 22.9 months, respectively (p=0.002).(Figure 2).

## DISCUSSION

It has been confirmed that the category based on the number of metastatic lymph nodes is more sensitive than that based on the location in prognostic evaluation of gastric cancer, especially when a minimum of 15 lymph nodes are examined in the surgical specimen [14]. Since 1997 the MLR has been showed to be a good alternative to prevent the stage migration phenomena and predict prognosis in a series of studies [8-13]. However, there was still no consensus about the cut-off limit for MLR in these studies. Many of these studies designed a 0.2 to 0.3 cut-off for MLR and reported patients with higher MLR had significantly lower survival rate. In our study, the appropriate MLR intervals were determined by the log-rank test. Consistent with these results, the high MLR was a poor prognostic factor for stage III gastric cancer patients and it remained as an independent factor according to the results of multivariate analysis. The median OS of MLR0 to MLR3 was 37.1m, 35.9m, 31.5m and 20.8m, respectively (p=0.013). It has seemed that a cut off level of 0.1 and 0.25 was suitable for categorizing MLR in stage III gastric cancer patients.

Two large phase III trials showed the benefit of adjuvant chemotherapy for gastric cancer, including stage III gastric cancer patients [15, 16]. Which patients benefit more? Could MLR be predictive factor of the efficacy of chemotherapy? There was no related study before. Our results showed MLR to be an appropriate factor to evaluate the benefit from adjuvant chemotherapy. For the patient with high MLR (MLR>0.24), median OS was significantly longer in the patients with adjuvant chemotherapy than without chemotherapy (22.9m vs 12.2m, p=0.002), which further indicated the additional value of MLR in helping screen the patients with high risk of recurrences or metastases who needed systemic therapy most.

A possible limitation of the present study might be the relative small number of patients analyzed. Thus, larger-scale study is needed to confirm the results. Furthermore, to illustrate the value of MLR in prognosis and stage, comparison of MLR and TNM stage system involving large-scale population is necessary.

In summary, the results of this study indicated that the MLR might act as a simple and effective indicator for prognostic factor for stage III gastric cancer after D2 nodal dissection. Furthermore, high MLR may benefit most from adjuvant chemotherapy.

## MATERIALS AND METHODS

This study enrolled 87 gastric cancer patients who underwent D2 lymphadenectomy at Zhejiang Cancer Hospital from December 2010 to March 2014. The pathology was gastric adenocarcinoma. The clinical and pathological information has been extracted. The records with ambiguous data were excluded.

Lymph node stage was classified according to the 2010 American Joint Committee on Cancer (AJCC) staging criteria for all patients. MLR was defined as number of metastatic lymph nodes divided by total resected lymph nodes. MLR intervals were determined using the log-rank test.

The follow-up of the patients was conducted with the following endpoints: disease-free survival (DFS, time from diagnosis to the first recurrence of the disease after the surgical treatment or death), and overall survival (OS, time from diagnosis to all causes of death).

Survival analysis and curves were established according to the Kaplan-Meier method and compared the log-rank test. Univariate and multivariate analyses were carried out by the Cox proportional hazards model. In all statistical analyses, a P value of <0.05 was considered significant. Data analysis was carried out with the Statistical Package for Social Sciences (SPSS) version 13.0.
